# Kinetic and Spectroscopic Studies of Bicupin Oxalate Oxidase and Putative Active Site Mutants

**DOI:** 10.1371/journal.pone.0057933

**Published:** 2013-03-01

**Authors:** Ellen W. Moomaw, Eric Hoffer, Patricia Moussatche, John C. Salerno, Morgan Grant, Bridget Immelman, Richard Uberto, Andrew Ozarowski, Alexander Angerhofer

**Affiliations:** 1 Department of Chemistry and Biochemistry, Kennesaw State University, Kennesaw, Georgia, United States of America; 2 Foundation for Applied Molecular Evolution, Gainesville, Florida, United States of America; 3 National High Magnetic Field Laboratory, Florida State University, Tallahassee, Florida, United States of America; 4 Department of Chemistry, University of Florida, Gainesville, Florida, United States of America; Max Planck Institute for Polymer Research, Germany

## Abstract

*Ceriporiopsis subvermispora* oxalate oxidase (CsOxOx) is the first bicupin enzyme identified that catalyzes manganese-dependent oxidation of oxalate. In previous work, we have shown that the dominant contribution to catalysis comes from the monoprotonated form of oxalate binding to a form of the enzyme in which an active site carboxylic acid residue must be unprotonated. CsOxOx shares greatest sequence homology with bicupin microbial oxalate decarboxylases (OxDC) and the 241-244DASN region of the N-terminal Mn binding domain of CsOxOx is analogous to the lid region of OxDC that has been shown to determine reaction specificity. We have prepared a series of CsOxOx mutants to probe this region and to identify the carboxylate residue implicated in catalysis. The pH profile of the D241A CsOxOx mutant suggests that the protonation state of aspartic acid 241 is mechanistically significant and that catalysis takes place at the N-terminal Mn binding site. The observation that the D241S CsOxOx mutation eliminates Mn binding to both the N- and C- terminal Mn binding sites suggests that both sites must be intact for Mn incorporation into either site. The introduction of a proton donor into the N-terminal Mn binding site (CsOxOx A242E mutant) does not affect reaction specificity. Mutation of conserved arginine residues further support that catalysis takes place at the N-terminal Mn binding site and that both sites must be intact for Mn incorporation into either site.

## Introduction

**Figure 1:**
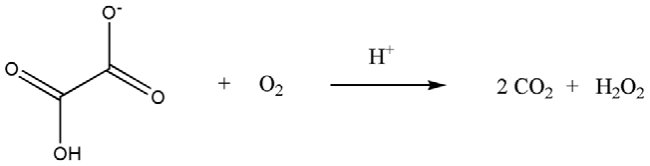
The reaction catalyzed by *Ceriporiopsis subvermispora* oxalate oxidase.

Oxalate oxidase (OxOx, E.C. 1.2.3.4), a manganese dependent enzyme, catalyzes the oxygen-dependent oxidation of oxalate to carbon dioxide in a reaction that is coupled with the formation of hydrogen peroxide (Figure 1) [1], [2]. This enzyme is of interest to a variety of commercial applications such as: determination of oxalate levels in blood and urine [3], [4], the protection of plants against pathogens, the production of transgenic plants with reduced levels of oxalate [5], [6], the bioremediation of oxalate waste, the production of hydrogen peroxide, and pulping in the paper industry [5], [7], [8], [9]. Moreover, OxOx is also interesting at a fundamental level, for the unique chemistry it catalyzes [10].

**Figure 2:**
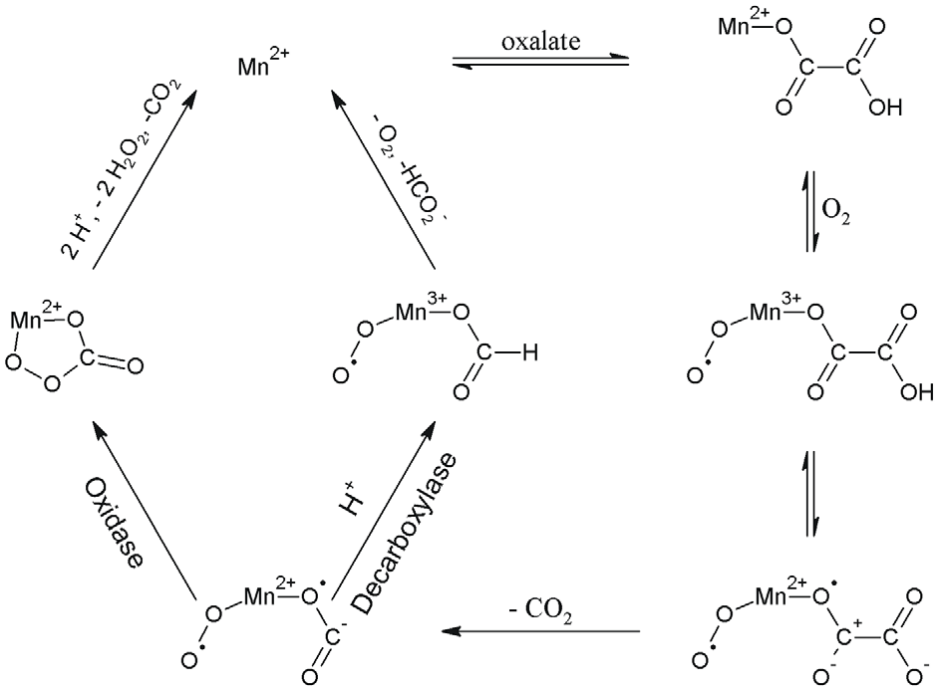
Model of free-radical mechanisms for oxalate oxidase and oxalate decarboxylase. Modified from [23].

**Figure 3:**
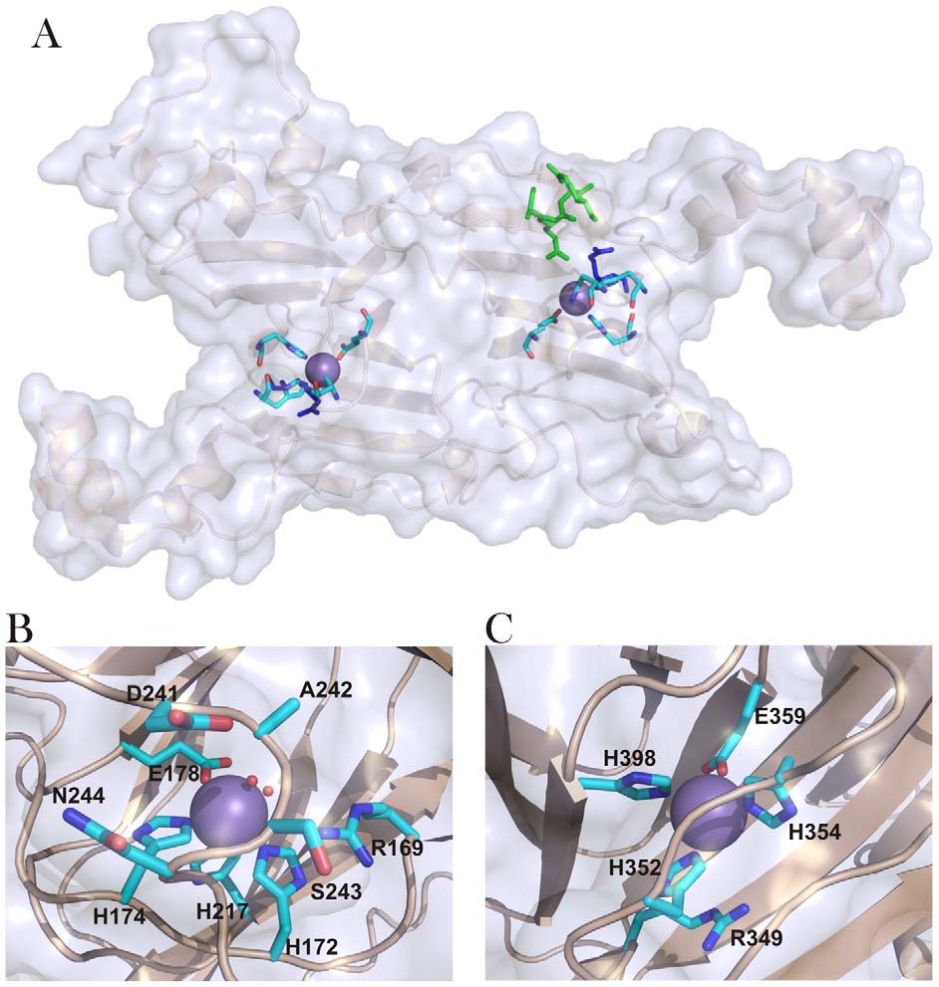
Manganese binding sites of the oxalate decarboxylase monomer and homology models of the manganese binding sites of CsOxOx. (A) OxDC (PDB ID 1UW8) [32] with manganese ions (purple), metal coordinating residues (atoms colored as follows: C, cyan; N, blue; O, red), conserved active site arginine residues (dark blue) and the N-terminal lid region (green) highlighted. (B) Homology model of the N-terminal CsOxOx Mn binding site metal coordinating residues and the DASN of the lid region. (C) Homology model of the C-terminal CsOxOx Mn binding site metal coordinating residues. The homology model of CsOxOx was constructed using its amino acid sequence and the experimentally solved structure of *Bacillus subtilis* OxDC (PDB ID 1UW8) using Swiss-Model (The Swiss Institute of Bioinformatics) [48], [49], [50]. Figure generated using Pymol (The PyMOL Molecular Graphics System, Schrödinger, LLC).

OxOx activity has been detected in wheat [11], barley [1], [12], [13], beet [14], [15], sorghum [16], [17], maize, oats, rice, and rye [10], [18]. Fungal OxOx activity was first reported in *Ceriporiopsis subvermispora*, a white rot basidiomycete fungus able to degrade lignin [19]. Plant OxOx enzymes have been structurally characterized and classified as monocupins [5], [20], [21], [22]. In the absence of structural data, prior homology modeling studies predicted that the *C. subvermispora* enzyme (CsOxOx) is the first manganese-containing bicupin enzyme identified that catalyzes oxalate oxidation [23]. CsOxOx shares greatest sequence identity (49%) with bicupin microbial oxalate decarboxylases (OxDC), which catalyzes the carbon-carbon bond cleavage of oxalate to yield carbon dioxide and formate (Figure 2) [23], [24], [25]. OxDC (and by homology, CsOxOx) is composed of two nearly structurally equivalent β-barrel domains each containing a manganese ion coordinated by four conserved amino acid residues (3 histidine and 1 glutamate) (Figure 3).

The proposed mechanisms for OxOx and OxDC involve the binding of oxalate directly to Mn(II), the formation of Mn(III), and a radical intermediate species (Figure 2) [2], [10], [26], [27]. Decarboxylation is facilitated by a reversible proton-coupled electron transfer that yields a manganese-bound formyl radical. EPR spin-trapping experiments support the existence of an oxalate-derived radical species formed during turnover [28]. Conversely, in OxDC from *Bacillus subtilis*, an active site glutamic acid (Glu162 in the N-terminal domain or Glu333 in the C-terminal domain) is proposed to protonate the manganese-bound formyl radical before formate is released.

Previously, we have shown that CsOxOx activity directly correlates with Mn content, and other metals do not appear to be able to support catalysis [28]. EPR spectra indicate that the Mn is present as Mn(II), and are consistent with the coordination environment expected from homology modeling with known X-ray crystal structures of OxDC from *Bacillus subtilis*. The pH dependence of this reaction suggests that the dominant contribution to catalysis comes from the monoprotonated form of oxalate binding to a form of the enzyme in which an active site carboxylic acid residue must be unprotonated [28].

It has been noted that OxDC's and OxOx's possess a small but measurable amount (<1%) of the other respective activity [23], [29]. An E162Q mutant of OxDC exhibited an approximately 200-fold decrease in OxDC activity with an approximately 10-fold increase in OxOx activity [30], a specificity switch of 2,000. Moreover, a theoretical study by Chang *et al*. suggested that the intrinsic reactivity of the N-terminal Mn-binding site of OxDC is to oxidize oxalate and that decarboxylation is a result of the protein environment modulating this reactivity through the addition of a proton donor as well as through electrostatic changes [31]. Burrell *et al*. have demonstrated that the decarboxylase can be converted to an oxidase by mutating amino acids of a flexible lid that, when in the “closed” conformation, seals the N-terminal Mn-binding site from bulk solvent [27]. The C-terminal Mn-binding site of OxDC does not possess an obvious lid region equivalent to that of the N-terminal site [32]. Mutations of this lid, which contains Glu162, have resulted in specificity switches of up to 282,000 (SEN161-3DSS), 275,000 (SENS161-4DSSN), and 225,000 (SENS161-4DASN). The structure of the SENS161-4DSSN mutant showed that the C-terminal Mn-binding site was not affected. This group's sequence information of oxalate oxidase from *C. subvermispora* played a critical role in the recognition that lid peptide sequences can control reaction specificity. Recombinant CsOxOx possesses less than 0.1% OxDC activity, consistent with the observation that the enzyme purified from *C. subermispora* possesses less than 0.2% OxDC activity [23]. By characterizing the active site of oxalate oxidase from *C. subvermispora* we can enhance the understanding of how subtle structural changes result in remarkable functional variation in evolutionarily related proteins.

## Materials and Methods

### Materials

Unless otherwise stated, all chemicals and reagents were purchased from Sigma-Aldrich and were of the highest available purity. All DNA primers were obtained from Integrated DNA Technologies, Inc., and confirmatory DNA sequencing was performed by the Georgia Genomics Facility at the University of Georgia. Restriction enzymes and other molecular biology reagents were purchased from New England Biolabs. Protein concentration was determined using a modified Lowry assay (Pierce) using bovine serum albumin as a standard [33].

### Expression and purification of “untagged” recombinant wild type CsOxOx and site-specific CsOxOx mutants

The expression and purification of recombinant wild type oxalate oxidase as a secreted protein using a *Pichia* expression system was carried out as previously described [28]. All site specific CsOxOx mutants were constructed using the overlap extension method [34] and the pPICZαA plasmid (Invitrogen) containing the gene encoding CsOxOx [28] (GenBank Accession Number: AJ746412). Primers for mutagenesis are provided in Supporting Information Table S1. Primers include restriction sites to facilitate cloning into pPICZαA and sites to aid the selection of transformants. The regions upstream and downstream of the mutagenesis site were amplified independently, and a third reaction combined these fragments to yield the full-length gene. The resulting products were digested with XhoI and XbaI and cloned into pPICZαA. Constructs were transformed into NEB 5-alpha competent *E. coli* cells (New England Biolabs) and transformants were screened by restriction digests. Plasmids containing the desired clones were transformed into X-33 competent cells (Invitrogen), and the expression of the CsOxOx mutants in *Pichia pastoris* was carried out as for the wild type enzyme [28].

### Steady – state kinetic assays

The level of oxalate oxidase activity was determined using a continuous assay in which H_2_O_2_ production is coupled to the horseradish peroxidase (HRP) catalyzed oxidation of 2,2′-azinobis-(3-ethylbenzthiazoline-6-sulphonic acid) (ABTS) [13]. All reactions were carried out at room temperature (25°C). Reaction mixtures contained 25 U HRP, 5 mM ABTS, 50 mM potassium oxalate, CsOxOx (at concentrations up to 0.050 mg/mL) dissolved in 50 mM sodium succinate, pH 4.0 (total volume 1.0 mL). Assays were monitored at 650 nm for 40 s and an extinction coefficient of 10,000 M^−1^ cm^−1^ for the ABTS radical product was assumed in these experiments. Control samples omitted HRP in order to distinguish between H_2_O_2_ production and any oxalate-dependent dye oxidation activity by CsOxOx. Measurements were made at specific substrate and enzyme concentrations in duplicate, and data were analyzed to obtain the values of V_max_ and V_max_/K_m_ by standard computer-based methods.

The pH dependence of V_max_/K_m_ was determined by measuring the initial rate at varying concentrations of oxalate. A multicomponent buffer system was used to provide a constant ionic strength of 150 mM and to minimize salt effects. Assay mixtures in these experiments each contained a final concentration of 50 mM glycine, 50 mM succinate, and 50 mM MES adjusted to specific pH values. Substrate solutions were pH adjusted also. Product formation (initial velocity) was linear over the course of the experiment for all pH values tested. The log(V_max_/K_m_) and log(V_max_) were plotted versus pH, and the data were fit to equations 1 and 2 using the program Excel 2007 (Microsoft Corporation).
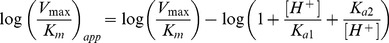
(1)

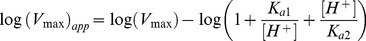
(2)


In addition to measuring the rate at which hydrogen peroxide was produced by the enzyme-catalyzed oxidation of oxalate, we determined the level of oxalate decarboxylase activity of CsOxOx. Assay mixtures consisted of 50 mM NaOAc, pH 4.2, 0.2% Triton X-100, 0.5 mM *o*-phenylenediamine, 1–50 mM potassium oxalate, and CsOxOx (50 μM) (100 μL total volume). Reactions were initiated by the addition of substrate, incubated at ambient temperature (21–23°C) with reactions times varying from 2 to 60 minutes, and quenched by the addition of 1 N NaOH (10 µL). The amount of formate produced was determined by an end-point assay [35] consisting of 50 mM potassium phosphate, pH 7.8, 0.09 mM NAD^+^, and 0.4–1.0 U/mg of formate dehydrogenase (1 mL final volume). The absorbance at 340 nm was measured after incubation at 37°C for 14 hours, and formate was quantified by comparison to a standard curve generated by spiking pre-quenched assay mixtures with known amounts of sodium formate.

### Determination of Mn content

The metal content of CsOxOx samples was quantified at the University of Georgia Center for Applied Isotope Studies Chemical Analysis Laboratory on the basis of inductively-coupled plasma mass spectroscopy (ICP-MS) [36]. To prepare samples and blanks for determination of metal content, divalent cations were removed from the final enzyme storage buffer by passing through a 1.5×16 cm column containing Chelex 100 (Bio-Rad) in the Na^+^ form. Purified protein samples were exchanged into the resulting buffer by washing 2.5 mg samples three times with 10-fold volumes of the “scrubbed” buffer in Centricon or Centriprep 30 (Amicon) concentrators [37]. The final filtrates recovered were used as blanks, which possessed insignificant metal content.

### Circular Dichroism (CD) Studies

All the CD experiments were carried out using a JASCO J-710 Spectropolarimeter (JASCO Inc., Tokyo, Japan) at wavelengths over the range of 195–250 nm in a 0.1-cm path length cell with a response time of 8 s and a scan speed of 50 nm/s. Each data point was an average of three accumulations. All samples analyzed were dialyzed into 25 mM potassium phosphate (pH 7.0) containing 25 mM NaCl and the protein concentration was adjusted to a final concentration of 938 μg/mL. All spectra were corrected by subtracting the CD spectrum of the buffer over the same range of wavelengths.

### EPR spin-trapping experiments

The reaction mixtures, conducted in 0.2 M potassium acetate buffer pH 4.1, contained: 100 mM oxalate, 100 mM KCl, 20 mM phenyl-N-t-butylnitrone (PBN) or 50 mM 5,5-dimethylpyrroline- N-oxide (DMPO), and 50 μM CsOxOx. A small aliquot of the reaction mixture was transferred into a quartz capillary of approximately 1×3 mm ID×OD for EPR measurement. The EPR spectrum was recorded at room temperature, using a Bruker Elexsys E580 X-band spectrometer, employing the Bruker high-Q cavity (ER 4123SHQE). EPR parameters used were typically: 100 kHz modulation frequency, 1 G modulation amplitude, 20.18 ms time constant, 81.92 ms conversion time/point, 70 G sweep width, 9.87 GHz microwave frequency, and 2 mW microwave power.

### Low-Temperature X-band EPR spectroscopy

EPR spectra were determined using CsOxOx (16 mg/mL) in 25 mM Imidazole-Cl, pH 7.0 (100 μL total volume). The samples were added to a 3×4 mm (IDxOD) quartz tube (approx. 100 μL per sample) then quickly frozen in pre-cooled isopentane. A test tube of liquid isopentane was inserted into a dewar filled with liquid nitrogen. After 30 to 40 s as the isopentane started to freeze at the walls of the test tube the EPR sample tube was plunged into the cold isopentane, which resulted in immediate freezing of the sample inside. When preparing samples at lower pH in acetate buffer a 1.0 M acetate buffer stock solution was prepared and adjusted to pH 4.0 using HCl. For oxalate addition we prepared a 0.5 M oxalic acid solution using KOH to adjust the pH to 4.0. 15 μL of the acetate buffer stock solution was added to 150 μL of CsOxOx enzyme solution for a sample at low pH (measured as 4.6) and a final acetate concentration of about 91 mM. To this sample 18 μL of the oxalate stock solution was added for a final oxalate concentration of about 50 mM. The sample was then frozen as described above in pre-cooled isopentane after a reaction time of about 5 seconds. EPR experiments at cryogenic temperatures were conducted on a Bruker Elexsys E580 spectrometer equipped with an Oxford ESR900 helium cryostat using the Bruker standard rectangular TE102 cavity. Pre-frozen samples were inserted into the pre-cooled cryostat. The instrumental settings for the spectra shown here were: sweep width, 7,000 G; number of data points/sweep, 2048; microwave frequency, 9.454 GHz; microwave power, 630 μW; modulation amplitude, 20 G; modulation frequency, 100 kHz; receiver gain, 70 dB; time constant, 40 ms; conversion time, 40 ms, temperature, 5 K, and each spectrum was the average of four field sweeps.

### High Field EPR Spectroscopy

High-Field EPR spectra were recorded at variable frequency/field combinations between 100 GHz/3 T and 400 GHz/15 T at the National High Magnetic Field Laboratory (NHMFL) in Tallahassee/FL using a home-built homodyne transmission-mode spectrometer with a non-resonant probe [38]. Sample preparation was performed the same way as for X-band EPR. Approx. 200 μL of sample was placed in a Teflon cup and inserted into the oversized waveguide at the point of highest homogeneity of the 17 T superconductive magnet. Typical instrument settings were: 45 kHz modulation frequency, 13 G modulation amplitude for low-resolution scans and 1 G modulation amplitude for high resolution scans.

## Results

### Enzyme expression and purification of the “untagged” site-specific CsOxOx mutants

A series of CsOxOx mutants were prepared and heterologously expressed in *Pichia pastoris* using a protocol developed in our laboratory [28] that yields wild type enzyme containing 0.4 Mn/monomer with a specific activity of 12 to 18 U/mg. There have been no metal content determinations reported for the native enzyme. Since the specific activities of our recombinant wild type CsOxOx preparations show a direct correlation with Mn content we conclude that the specific activity of our preparations is limited by the amount of Mn that we can incorporate using our *Pichia* expression system [28]. Furthermore, since the specific activity of the recombinant, wild type enzyme compares favorably with that of the native enzyme [19], we conclude that though our Mn incorporation is relatively low it fairly represents the Mn occupancy of the native enzyme. Additionally, we have shown that other metals tested (Mg^2+,^ Co^2+^, Zn^2+^, Ni^2+^, and Fe^2+^) do not support catalysis [28]. Efforts to increase the manganese content of CsOxOx through reconstitution have not been successful. The cell growth, extracellular secretion, and chromatography steps (anion exchange and butyl-sepharose) of the purification proceeded similarly for the active site mutants as for the wild type enzyme.

### Steady – state kinetic characterization of the site-specific CsOxOx mutants

Of the six CsOxOx mutants prepared and assayed for catalytic activity, only the D241A and A242E mutants possessed measurable activity (Table 1). The manganese content was determined for all CsOxOx mutants and the V_max_ and oxalate K_M_ values were determined for the D241A and A242E mutants. The observation that the D241A mutant contains 17% of the Mn content of the recombinant wild type enzyme but only 7% of the wild type oxidase activity suggests that catalysis takes place at the N-terminal Mn-binding site. The K_M_ for oxalate binding in the D241A mutant is essentially unperturbed; thus the V_max_ and V_max_/K_M_ values when normalized are reduced to 40% and 30%, respectively, compared to the wild type CsOxOx. These data can be understood by either of two hypotheses: 1) Asp-241 is catalytically important, or 2) the observed Mn binding occurs at a catalytically inactive site. On the other hand, the replacement of Asp-241 to serine resulted in no detectable Mn binding or oxidase activity. This N-terminal mutation precluded the incorporation of manganese in either the N- or C-terminal domain.

**Table 1:** Steady state kinetic parameters and manganese content of recombinant, wild type CsOxOx and CsOxOx mutants.

	% Activity	V_max_, U/mg	K_M,_ mM^a^	Mn, mol/monomer	V_max_/[Mn], U/mg/mol Mn	V_max_/K_M_/[Mn], U/mg/mM/mol Mn
**Wild type**	100	12.8±0.02	0.8±0.1	0.36±0.01	35.6	44.4
**D241A**	7	0.87±0.04	1.1±0.2	0.06±0.01	14.5	13.2
**D241S**	<0.1	nd^b^	nd	<0.01		
**A242E**	5	0.62±0.04	2.0±0.4	0.15±0.02	4.1	2.1
**DASN241- 244SENS**	<0.1	nd	nd	<0.01		
**R169K**	<0.1	nd	nd	0.05±0.01		
**R349K**	<0.1	nd	nd	<0.01		

a K_M_ values were determined from assays in which oxalate concentration was varied over the range of 0.01 to 50 mM.

b nd. Value was not determined.

**Figure 4:**
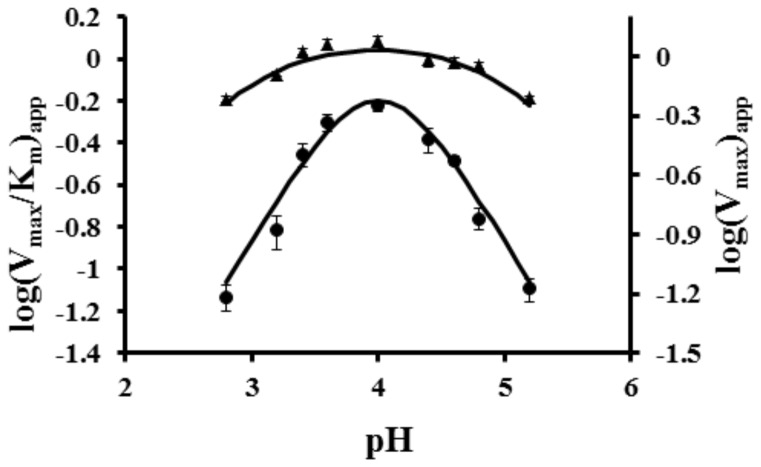
The effect of pH on the affinity of recombinant CsOxOx D241A for oxalate. pH dependence of CsOxOx D241A mutant on kinetic parameters V_max_/K_m_ (•) and V_max_ (▴) for the CsOxOx catalyzed reaction.

To aid in distinguishing the two hypotheses above, we analyzed the pH dependency of the D241A mutant enzyme (Figure 4). We have previously shown [28] that the pH profile of the recombinant wild type enzyme required at least three pK_a_ values (pK_a1_ = 4.5, pK_a2_ = 3.1, and pK_a3_ = 3.7) to approximate the data. The slope of the acidic limb was approximately +2 and the slope of the basic limb was approximately -1. On the other hand, the pH profile of the D241A mutant (Figure 4) could be fit with two pK_a_ values (pK_a1_ = 5.1 and pK_a2_ =  2.9). The slope of the acid limb is approximately +1 and the slope of the basic limb is approximately -1, suggesting that aspartic acid 241 contributes the third pK_a_ to the wild type pH profile and contributes significantly to catalysis.

The replacement of Ala-242 with a glutamate residue (A242E) was done to address whether OxDC activity could be introduced into the oxidase through the introduction of an active site proton donor. The A242E mutant, like the recombinant wild type enzyme, possessed <0.1% oxalate decarboxylase activity (data not shown). Active site Arg residues, which in OxDC have been proposed to stabilize an anionic intermediate in both the CsOxOx N- and C-terminal Mn binding sites, were conservatively mutated to lysine residues. If only the N-terminal domain is involved in catalysis, in the absence of interactions between two sites the activity of the R169K mutant should be decreased while that of the R340K mutant should be unaffected. Mutation of Arg 169 reduces activity (<0.01% of the wild type enzyme) below that expected due to its level of Mn incorporation (14% of the wild type enzyme). This further supports the conclusion that catalysis takes place at the N-terminal Mn binding site, but this conclusion is slightly weakened by the results for R349K CsOxOx mutant. Mutation of Arg 349 abolishes Mn incorporation into either the N- or the C-terminal Mn binding site again suggesting that both Mn binding sites must be intact for Mn incorporation into either site.

### Circular dichroism (CD) measurements

**Figure 5:**
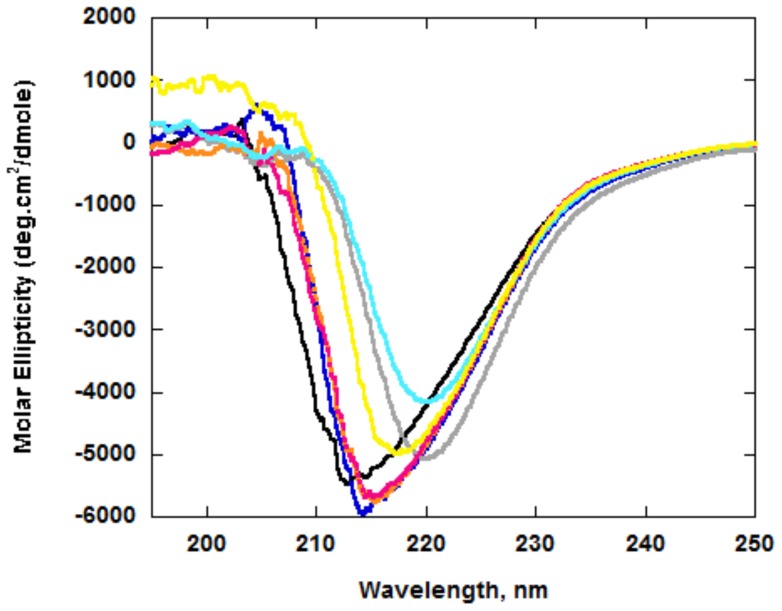
CD spectra of recombinant, wild-type CsOxOx and the putative active site mutants. All samples were 938 ug/mL in phosphate buffer (pH 7.0): wild-type CsOxOx, black; D241A, dark blue; D241S, grey; A242E, orange; DASN241-244SENS, red; R169K, cyan; R349K, yellow.

Circular dichroism (CD) measurements were carried out to monitor the degree of secondary structural changes due to the site-specific replacement of the putative active site residues under study. The CD spectrum of recombinant, wild type CsOxOx (Figure 5) exhibited a single minimum at 213 nm which is consistent with expectations for a protein composed primarily of β-strands. Three of the six CsOxOx mutants (D241A, A242E, and DASN241-244SENS) showed spectra that closely resemble the spectra of the wild type enzyme with a single minimum at 214, 215, 215 nm, respectively. These data suggest that the global protein folding of the D241A, A242E, and DASN241-244SENS CsOxOx mutants was not disrupted relative to the wild type protein. CsOxOx mutants D241S, R169K, and R349K also displayed a single minimum (220, 220, and 217 nm, respectively) in the region associated with β-strand character but with decreased molar ellipticity values suggesting that these mutations had greater impact on the overall bicupin architecture of these proteins.

### X-band spin trapping experiments

**Figure 6:**
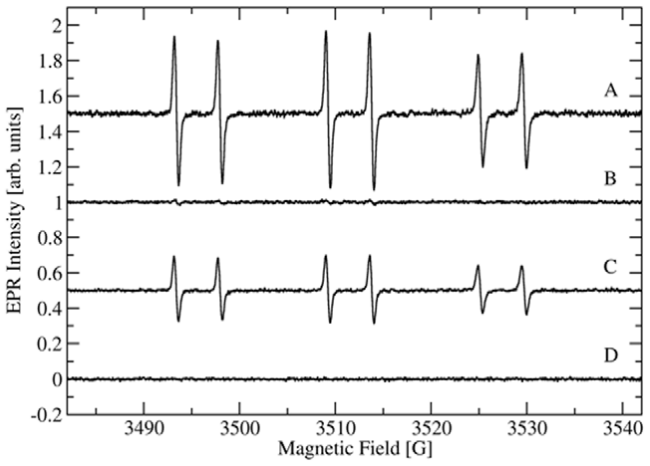
EPR spectra of PBN radical adducts obtained from the incubation of 100 **mM oxalate, 100** **mM KCl, 20** **mM PBN, and 50** **µM CsOxOx.** (A) PBN-oxalate derived radical adduct from the recombinant, wild type CsOxOx catalyzed reaction. (B) PBN-oxalate derived radical adduct from the CsOxOx A242E mutant catalyzed reaction showed no signal over background. (C) PBN-oxalate derived radical adduct from the CsOxOx D241A mutant catalyzed reaction. (D) All reaction components without enzyme.

The X-band EPR spectra of spin trapped free radicals produced during turnover by recombinant wild type and mutant CsOxOx enzymes are shown in Figure 6. Figure 6A shows the six-line spectrum of the PBN radical adduct formed during turnover. The hyperfine coupling constants are consistent with those of the PBN-CO_2_^ ˙−^ radical adduct [39]. These data strongly support the formation of the carbon dioxide radical as an intermediate in the proposed catalytic mechanism (Figure 2). Trapping of the same species in oxalate decarboxylase [39] is consistent with a carbon dioxide radical intermediate formed during turnover in both OxDC and CsOxOx (Figure 2). Figure 6B shows that barely any PBN radical adduct is formed during turnover of the A242E mutant. Figure 6C shows the sextet of the PBN radical adduct formed during turnover with the D241A CsOxOx mutant. Finally, Figure 6D shows the spin trapping control (experimental background without oxalate). Mutants that produce 5 to 7% of wild-type activity can have significant detectable carboxylate radical intermediate (D241A) or none at all (A242E). This may reflect perturbation of different steps in the catalytic cycle, and might also reflect side paths that make the carboxylate radical more inaccessible to the spin trap. This is consistent with the current view of enzyme catalysis in which cooperative conformational changes and multiple reaction intermediates are prevalent in the mechanisms of enzymes [40]. The observation of two conformations of OxDC in the crystal structures taken at high pH [32], [41] and the demonstration of the importance of its “lid” region [27] is consistent with the view that the initial binding of substrate “closes” over the active site and drastically alters the environment, perhaps excluding water and altering pK_a_ values. The A241E CsOxOx mutant may cause a conformation to be populated significantly decreasing the accessibility of the radical to reach the spin trap.

### Multi-frequency EPR of the manganese centers of CsOxOx

**Figure 7:**
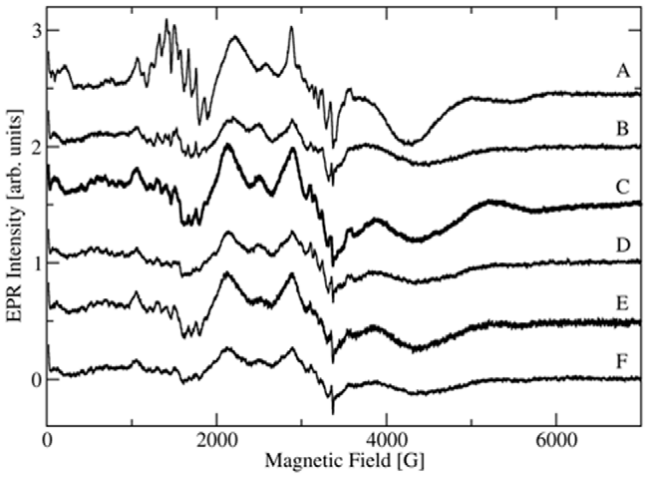
X-band EPR of the Mn(II) centers of wild type CsOxOx and CsOxOx A242E mutant under several different conditions at 5 **K.** 7A: wild type CsOxOx in imidazole buffer at pH 7.0, as taken from the original preparation. 7B: CsOxOx A242E mutant in imidazole buffer at pH 7.0, as taken from the original preparation. 7C: Same sample as 7A, thawed and after addition of 100 mM acetate buffer pH 4.0. 7D: Same sample as 7B, thawed and after addition of 100 mM acetate buffer pH 4.0. 7E: Same sample as in 7C thawed and after addition of 50 mM oxalate and allowed to further react for approximately 2 min. 7F: Same sample as in 7D thawed and after addition of 50 mM oxalate and allowed to further react for approximately 2 min.

A previous simulation [28] was able to reproduce quite well the main broad features of the X-band spectra using a single set of magnetic parameters (*D* = −3000 MHz, *E* = 300 MHz, *g* = 2.00088, *A* = 263 MHz with 20% strain associated with the fine structure constants *D* and *E*). In this contribution, a multi-frequency approach was used to obtain more precise data on the magnetic parameters. EPR spectra recorded at 20 K in the X-band (Figure 7), and at 104, 208, 326.4 and 416 GHz (Figure S2) were simulated using the easyspin package in Matlab [42]. All simulations are shown in Supporting Information (Figures S3–S7). While the simulations are not perfect, a consistent picture emerged that gave reasonable fits for the main peaks in both the high field spectra as well as in the central part of the X-band spectrum using only one set of magnetic parameters, *g*_iso_  = 2.00108, *A*_iso_  = 243 MHz, |*D*| = 2850 MHz, |*E*| = 280 MHz. The fit could probably be improved by assuming anisotropic *g*-factor and hyperfine coupling constants which seems reasonable given the relatively large zero field splitting parameters, which suggest a distorted octahedral environment for the primary Mn(II) site. Moreover, the region between 100 and 200 mT in the X-band suggests the presence of a second Mn(II) site, possibly a rhombic site with very large zero field splittings indicative of a pentacoordinated site. However, there was little indication of this in the high field spectra, which suffered from relatively poor signal-to-noise ratio and didn't allow us to determine the transitions between higher spin manifolds. As a result we decided to not simulate a second site further without additional experimental evidence.

**Figure 8:**
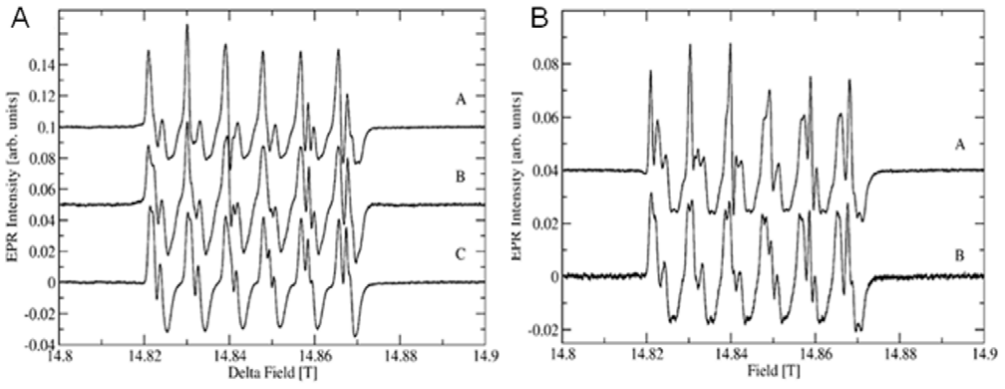
pH dependence of recombinant, wild-type CsOxOx and A242E CsOxOx mutant at 416.0 **GHz.** 8A: Recombinant, wild type CsOxOx A: Same sample as C after the addition of 100 mM glycine buffer, pH 3.0. B: Same sample as C after the addition of 100 mM acetate buffer, pH 4.0. C: in 25 mM Imidazole-Cl, pH 7.0. 8B: A242E CsOxOx mutant. A: Same sample as B after the addition of 100 mM acetate buffer, pH 4.0, B: in 25 mM Imidazole-Cl, pH 7.0.

The pH dependence of the wild type CsOxOx EPR spectra at X-band are shown in Figure 7 and at 416 GHz in Figure 8A. The pH dependence of the A242E mutant is shown in Figure 8B. The frequency dependence of the wild-type enzyme is shown in Supporting Information, Figure S2. The Mn is not released from the enzyme at low pH. The pH dependence of the high-field spectra show very little if any change unlike what has been observed in OxDC [43]. The biggest change is seen in the X-band spectra, where the sharp features between 100 and 200 mT diminish at low pH. If they indeed belong to a pentacoordinated Mn(II) site similar to the C-terminal domain in OxDC their disappearance could be due to a conversion into a hexacoordinated species at low pH just as in OxDC. However, the high-field spectra do not indicate the appearance of another species with small zero field splittings, which should be quite conspicuous. More likely, the site becomes somewhat unstructured and shows a broad distribution of zero field splittings that would be difficult to detect. The existence of two different Mn(II) sites in CsOxOx complicates the assignment of observed spectra to specific sites just as in OxDC. Given our inability to directly detect transitions between higher spin manifolds using the limited concentration range available in these preparations no further insights are currently possible. We are working on increasing the solubility of the enzyme to allow for direct detection of the higher spin manifolds at very low temperatures combined with the addition of small chelators that could potentially bind to the active site, producing better resolved EPR spectra.

## Discussion

Given that the pH dependence of the recombinant, wild type enzyme suggested that an active site carboxylate residue was involved in catalysis [28] and that the 241-244DASN region of the N-terminal Mn-binding domain (Figure 3 and Figure S1) was analogous to the 161-164SENS lid region of OxDC (shown in green in Figure 3), we prepared a series of mutants to probe this region. To evaluate the effect of removing the carboxylate group from this region, Asp-241 was replaced with alanine (D241A) and with serine (D241S) to mimic the analogous position in OxDC. To address whether OxDC activity could be introduced into the oxidase through the introduction of an active site proton donor, Ala-242 was replaced with a glutamate residue (A242E). Similarly, the complete replacement of the tetrapeptide was accomplished by the construction of the DASN241-244SENS CsOxOx mutant.

That the K_M_ for oxalate binding in the D241A mutant is essentially unperturbed and that the V_max_ and V_max_/K_M_ values when normalized are reduced to 40% and 30%, respectively, compared to the wild type CsOxOx suggests that Asp 241 is catalytically important. In a previous communication we concluded that the pH dependence of the activity must be due in part to the requirement that at least one carboxylic acid residue be unprotonated [28]; the data for D241A identifies this residue as responsible for part of the pH dependence. In addition, it is now clear that the oxalate/oxalic acid pK_a_ at 1.3 is too low to account for the pK_a_ near 3 in the mutant enzyme. It is likely that in addition to D241 a second carboxylic acid group on the enzyme must be unprotonated for activity. No combination of binding constants with an oxalic acid pK of 1.3 can reproduce the observed pH profile.

All of the parameters are derived by fitting the Michaelis-Menton expression to kinetic results over a range of conditions, and the entire data set can be described by the parameters we report. We are reluctant to make very detailed deductions about enzyme mechanism from steady state kinetic results; we hope to be able to extend our results in subsequent studies using single turnover kinetic experiments. Here, we indentify a particular protonation state of the enzyme and substrate as the parent of downstream obligatory intermediates. This doesn't identify the rate limiting step in the reaction, but it does place limits on it. King-Altman analysis [44] indicates that the protonation state identified must be part of the rapid equilibrium segment, or at least a rapid equilibrium segment that can readily become rate limiting at sub-optimal pH. During steady state turnover, net transition rates between all rapid reaction segments are equal, so those segments with slower rate constants tend to be more heavily populated. To observe pH effects on V_max_, it is necessary to populate unproductive protonation states that reduce the population of the states prior to the limiting step.

The proposed catalytic mechanism for OxOx (Figure 2) [2], [10], [26], [27] has been shown to contain a common intermediate, an oxalate-derived radical species formed during turnover, with the decarboxylase reaction [28], presumably a Mn-bound formyl radical. In OxDC from *Bacillus subtilis*, an active site glutamic acid (Glu162 in the N-terminal domain or Glu333 in the C-terminal domain) is proposed to protonate the manganese-bound formyl radical before formate is released. In the absence of an active site proton donor, the manganese-bound formyl radical has been proposed to cyclize into a percarbonate intermediate (Figure 2), leading to the release of the hydrogen peroxide and the second molecule of carbon dioxide [23], [26], [27], [29]. Despite the expectation that this cyclization would be very fast, we replaced Ala-242 with a glutamate residue (A242E) to address whether OxDC activity could be introduced into the oxidase through the introduction of an active site proton donor. The side chain of Ala-242 is directed tangentially to the Mn ion in the homology model (Figure 3B) and its replacement with the carboxylate side chain of glutamate was anticipated to place an ionizable proton within reasonable proximity of the putative active site. The A242E mutant (actually all of the CsOxOx mutants described in this paper), like the recombinant wild type enzyme, possessed <0.1% oxalate decarboxylase activity (data not shown). Merely providing the enzyme with an additional potential active site proton donor is insufficient to convert it to a decarboxylase. It is interesting to note that the C-terminal domain of CsOxOx contains a conserved glutamate residue (Glu-412) analogous to Glu-333 in the C-terminal domain of OxDC (Figure S1).

A question exists in the literature as to which Mn center in OxDC is involved in catalysis or whether both are catalytically active. Initial site-directed mutagenesis studies suggested that the C-terminal Mn-binding site was the sole or dominant site of catalysis [41]. Other evidence, however, supports the hypothesis that the N-terminal Mn-binding site of OxDC is the sole or major catalytic site and that the C-terminal Mn-binding site plays a purely structural role. This evidence includes mutagenesis and structural studies [27], [32], [45], kinetic isotope effect studies with mutant enzymes [30], and studies that used high-field EPR techniques to distinguish at least two distinct manganese species based on pH dependence and differential binding of formate to each site [46]. It is also possible that both manganese binding sites in OxDC can catalyze the decarboxylation reaction [47]. In OxDC, Arg92 in the N-terminal domain and Arg-270 in the C-terminal domain have been implicated in substrate binding and charge stabilization of the catalytic intermediate [32], [41]. Since both of these residues are conserved in CsOxOx (Arg169 and Arg349) (Figure S1), we constructed R169K and R349K CsOxOx mutants to address the question of which Mn-binding domain contributes to catalysis. The rationale for substituting these arginine residues with lysine residues was twofold: first, it was predicted that the substitution of a positively charged polar amino acid with another positively charged amino acid would likely maintain protein stability, and second, the analogous mutations in OxDC (R92K and R270K) were reported to provide mechanistic insights without a complete loss of protein function. If only the N-terminal domain is involved in catalysis, the activity of the R169K mutant should be decreased while that of the R349K mutant should be unaffected.

Substitution of the guanidine moiety of Arg 169 with the amino group of lysine decreased activity (<0.01% of the wild type enzyme) below that expected due to its level of Mn incorporation (14% of the wild type enzyme). This would imply that catalysis takes place at the N-terminal Mn binding site, but this implication is slightly weakened by the results for the R349K CsOxOx mutant. The substitution of Arg 349 with lysine abolishes Mn incorporation into either the N- or the C-terminal Mn binding site again suggesting that both Mn binding sites must be intact for Mn incorporation into either site. In other words, if Mn cannot be incorporated into the C-terminal Mn binding site, it cannot be incorporated into the N-terminal Mn binding site. While both of the CsOxOx arginine mutants (and the D241S mutant), like the wild type enzyme and other CsOxOx mutants described in this work, displayed CD spectra consistent with primarily β-strand secondary structure, their decreased molar ellipticity and slightly shifted minima reflect a perturbed secondary structure in these variants. Given that the Mn ions in CsOxOx (by homology with OxDC) are 26 Å apart from each other, one question becomes how a mutation at the C-terminal Mn binding site can profoundly affect metal loading at the N-terminal Mn binding site (or vice versa). We are interested to explore a structural basis for a possible interaction between the two Mn-binding sites in CsOxOx in our future work.

In conclusion, the pH profile of the D241A CsOxOx mutant suggests that the protonation state of aspartic acid 241 is mechanistically significant and that catalysis takes place at the N-terminal Mn-binding site. The observation that the D241S CsOxOx eliminates Mn-binding to both the N- and C- terminal Mn-binding sites suggests that both sites must be intact for Mn incorporation into either site. The introduction of a proton donor into the N-terminal Mn binding site (CsOxOx A242E mutant) does not affect reaction specificity. The CsOxOx R169K mutation reduces activity below that commensurate with its Mn content which further supports that catalysis takes place at the N-terminal Mn-binding site. The CsOxOx R349K mutation abolishes Mn incorporation into both the N- or the C-terminal Mn-binding sites, confirming that both sites must be intact for Mn incorporation into either site and precluding a conclusive assessment of whether the C-terminal Mn-binding site can mediate catalysis.

### Supporting Information

Primers used in the construction of wild type and mutant CsOxOx enzymes (Table S1), the sequence alignment of *Bacillus subtilis* OxDC and CsOxOx (Figure S1), the frequency dependence of wild-type CsOxOx and A242E CsOxOx mutant (Figure S2), and the results of the best fits obtained for simulations of the EPR spectra (Figures S3–S7). All simulations were performed with the EasySpin toolbox [42] in the MATLAB computing environment (The MathWorks, Natick, MA).

## Supporting Information

Figure S1 **Sequence alignment of CsOxOx and OxDC.** Sequence alignment of *Bacillus subtilis* oxalate decarboxylase (OxDC, PDB code: 1uw8) [32] and *Ceriporiopsis subvermispora* oxalate oxidase (CsOxOx) by the Clustal W method [51], [52]. Asterisks indicate identical residues, colons (:) indicate conservative substitutions, and periods (.) indicate semi-conservative substitutions. The conserved cupin motifs are shown (motif 1 in blue and motif 2 in red) in the two domains. The “lid” region is shown in green. The Mn-binding residues are underlined.
(TIF)

Figure S2 **Frequency dependence of recombinant, wild-type CsOxOx in 25** **mM Imindazole-Cl, pH** **7.0.** A: 104.0 GHz. B: 208.0 GHz. C: 326.4 GHz. D: 416.0 GHz.
(TIF)

Figure S3 **X-band EPR spectrum of wild-type CsOxOx enzyme in 25** **mM Imidazole-Cl, pH** **7.0.** The experimental and simulated spectra are displayed in blue and red, respectively.
(TIF)

Figure S4 **104.0** **GHz EPR spectrum of wild-type CsOxOx enzyme in 25** **mM Imidazole-Cl, pH** **7.0.** The experimental and simulated spectra are displayed in blue and red, respectively.
(TIF)

Figure S5 **208.0** **GHz EPR spectrum of wild-type CsOxOx enzyme in 25** **mM Imidazole-Cl, pH** **7.0.** The experimental and simulated spectra are displayed in blue and red, respectively.
(TIF)

Figure S6 **326.4** **GHz EPR spectrum of wild-type CsOxOx enzyme in 25** **mM Imidazole-Cl, pH** **7.0**. The experimental and simulated spectra are displayed in blue and red, respectively.
(TIF)

Figure S7 **416.0** **GHz EPR spectrum of wild-type CsOxOx in 25** **mM Imidazole-Cl, pH** **7.0.** The experimental and simulated spectra are displayed in blue and red, respectively.
(TIF)

Table S1 **Primers used in the construction of wild type CsOxOx and CsOxOx mutants.**
(DOCX)
