# Supplementary figures and images for: Kinetic and Spectroscopic Studies of Bicupin Oxalate Oxidase and Putative Active Site Mutants
Source: PLoS One. 2013 Mar 1;8(3):e57933. doi: 10.1371/journal.pone.0057933 (PMC3585803; doi:10.1371/journal.pone.0057933)

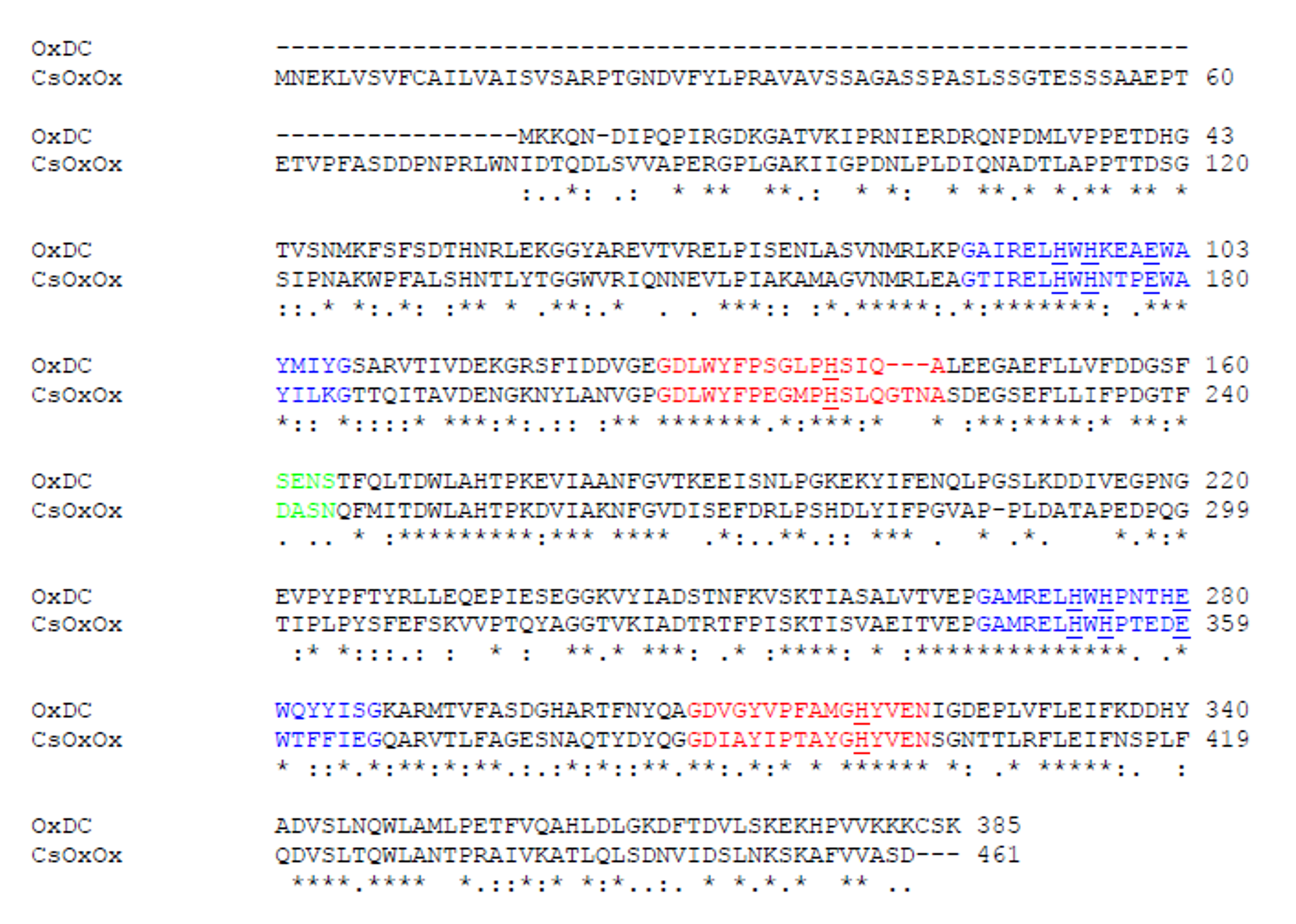

Supplement: Figure S1 — Sequence alignment of CsOxOx and OxDC. Sequence alignment of Bacillus subtilis oxalate decarboxylase (OxDC, PDB code: 1uw8) [32] and Ceriporiopsis subvermispora oxalate oxidase (CsOxOx) by the Clustal W method [51], [52]. Asterisks indicate identical residues, colons (:) indicate conservative substitutions, and periods (.) indicate semi-conservative substitutions. The conserved cupin motifs are shown (motif 1 in blue and motif 2 in red) in the two domains. The “lid” region is shown in green. The Mn-binding residues are underlined. (TIF) [file pone.0057933.s001.tif]

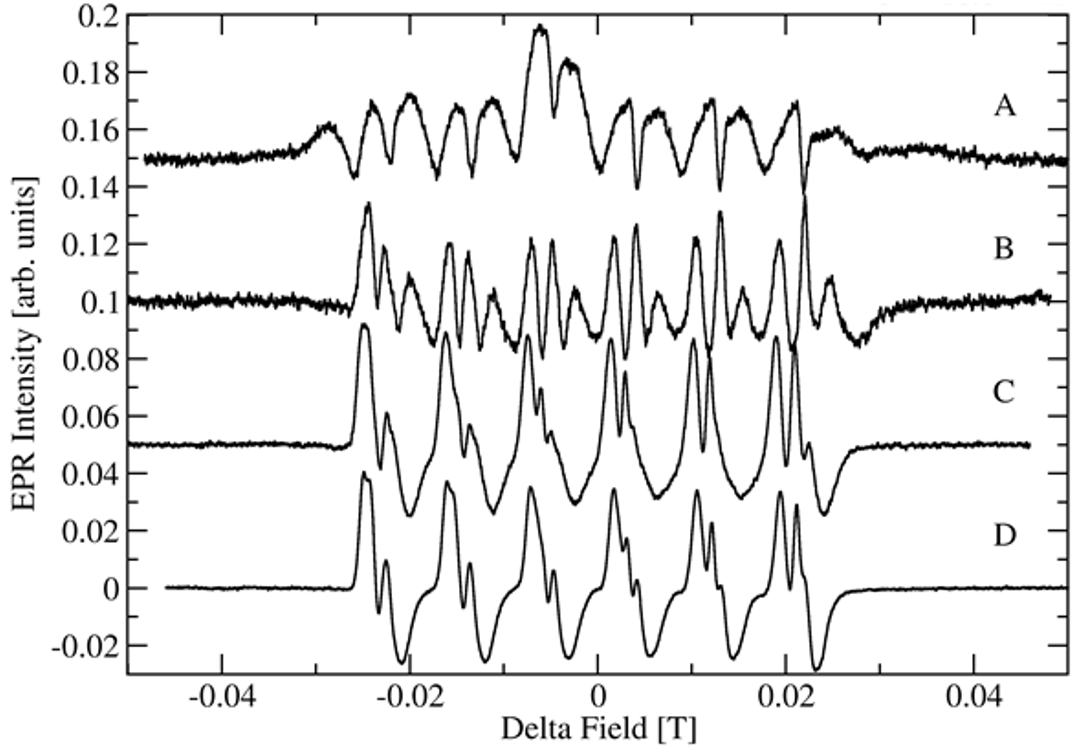

Supplement: Figure S2 — Frequency dependence of recombinant, wild-type CsOxOx in 25 mM Imindazole-Cl, pH 7.0. A: 104.0 GHz. B: 208.0 GHz. C: 326.4 GHz. D: 416.0 GHz. (TIF) [file pone.0057933.s002.tif]

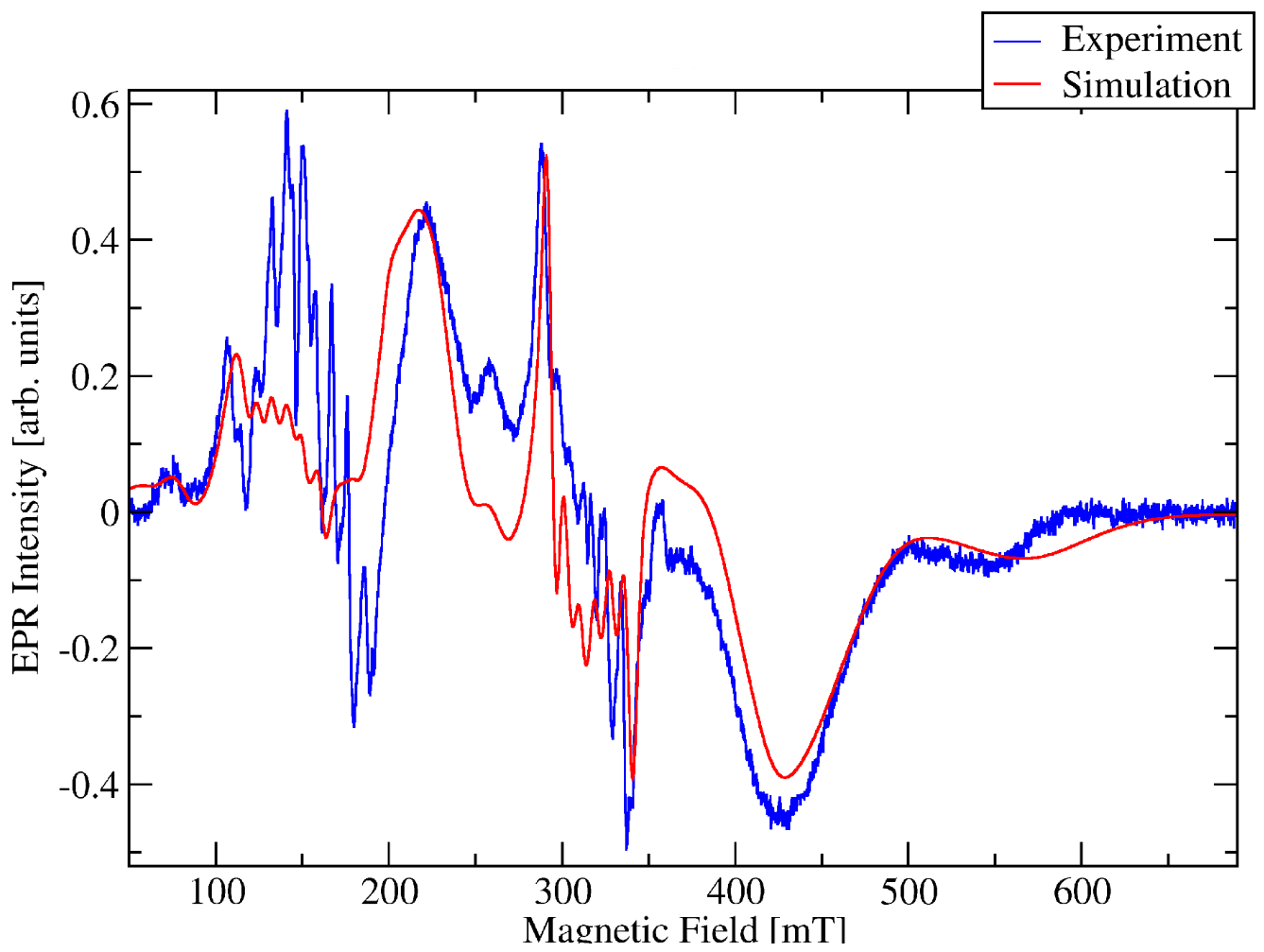

Supplement: Figure S3 — X-band EPR spectrum of wild-type CsOxOx enzyme in 25 mM Imidazole-Cl, pH 7.0. The experimental and simulated spectra are displayed in blue and red, respectively. (TIF) [file pone.0057933.s003.tif]

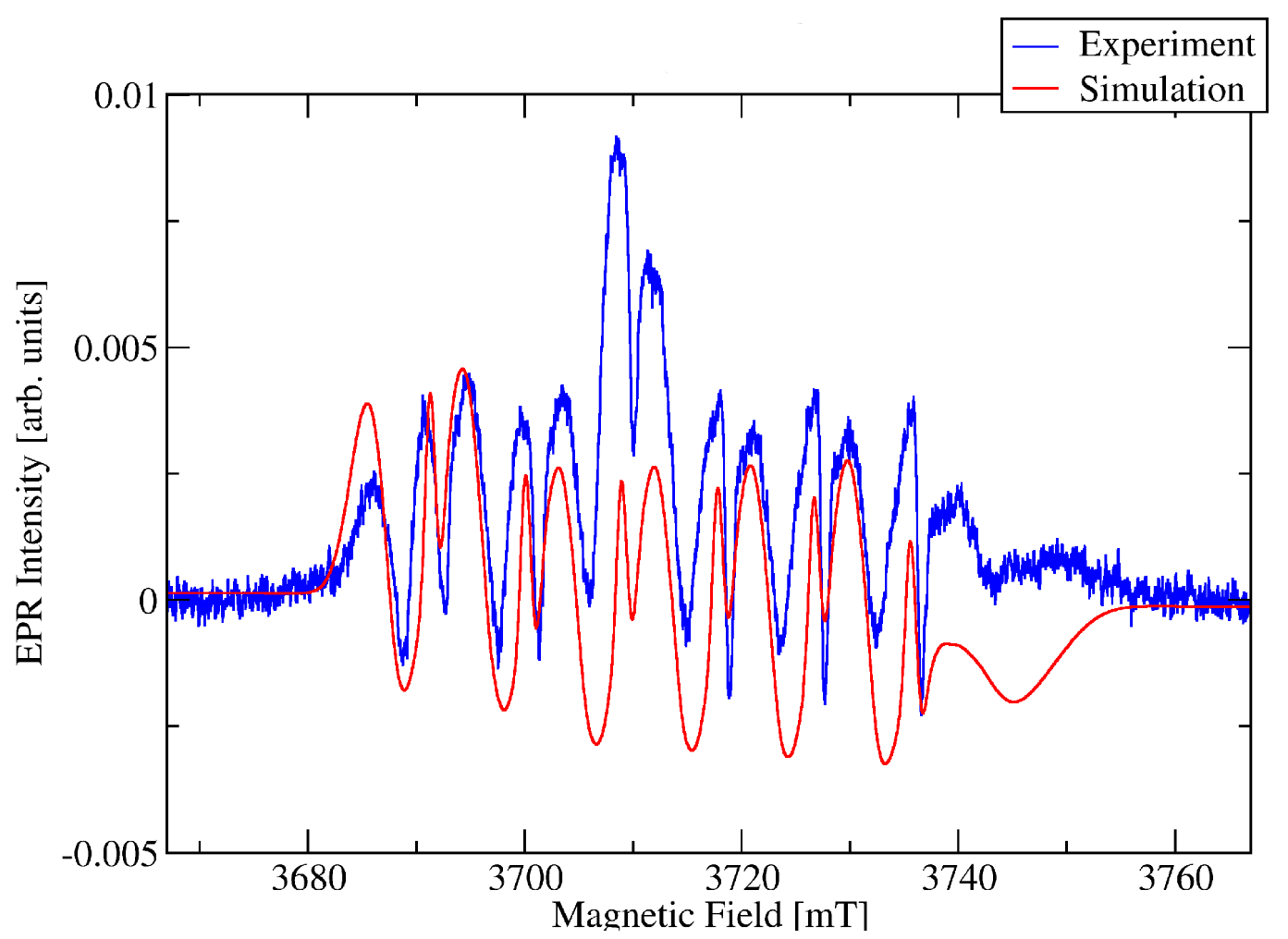

Supplement: Figure S4 — 104.0 GHz EPR spectrum of wild-type CsOxOx enzyme in 25 mM Imidazole-Cl, pH 7.0. The experimental and simulated spectra are displayed in blue and red, respectively. (TIF) [file pone.0057933.s004.tif]

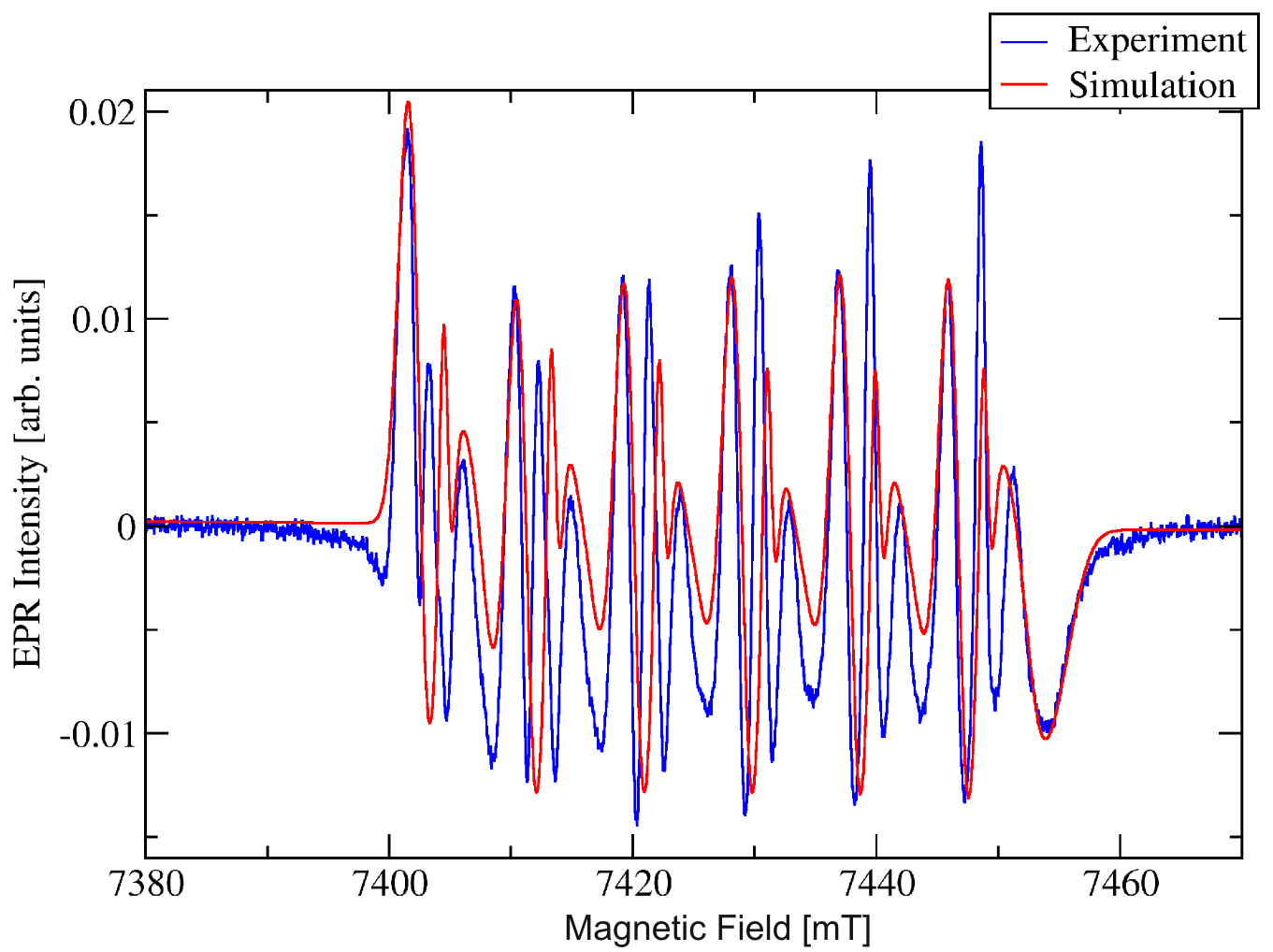

Supplement: Figure S5 — 208.0 GHz EPR spectrum of wild-type CsOxOx enzyme in 25 mM Imidazole-Cl, pH 7.0. The experimental and simulated spectra are displayed in blue and red, respectively. (TIF) [file pone.0057933.s005.tif]

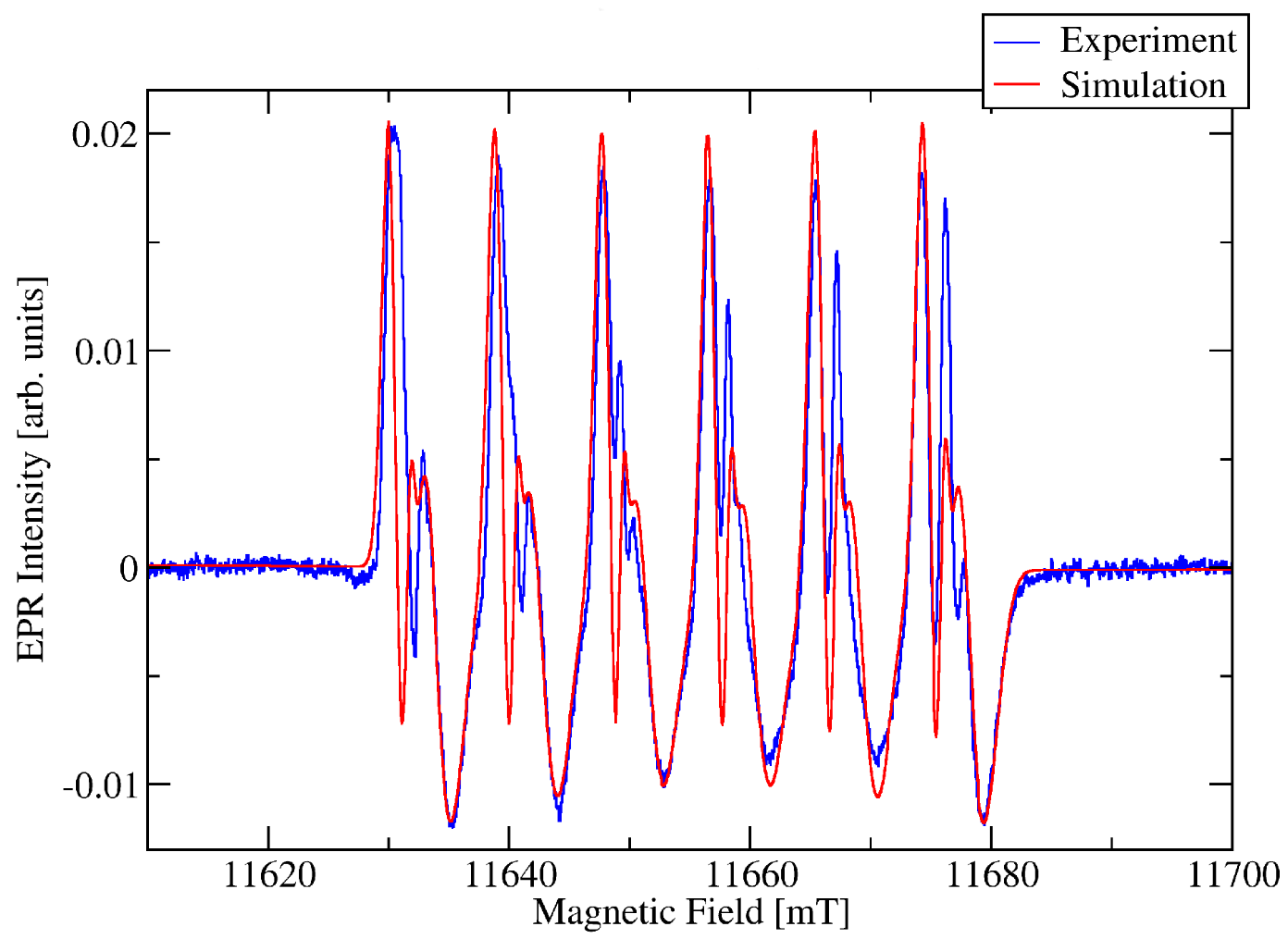

Supplement: Figure S6 — 326.4 GHz EPR spectrum of wild-type CsOxOx enzyme in 25 mM Imidazole-Cl, pH 7.0. The experimental and simulated spectra are displayed in blue and red, respectively. (TIF) [file pone.0057933.s006.tif]

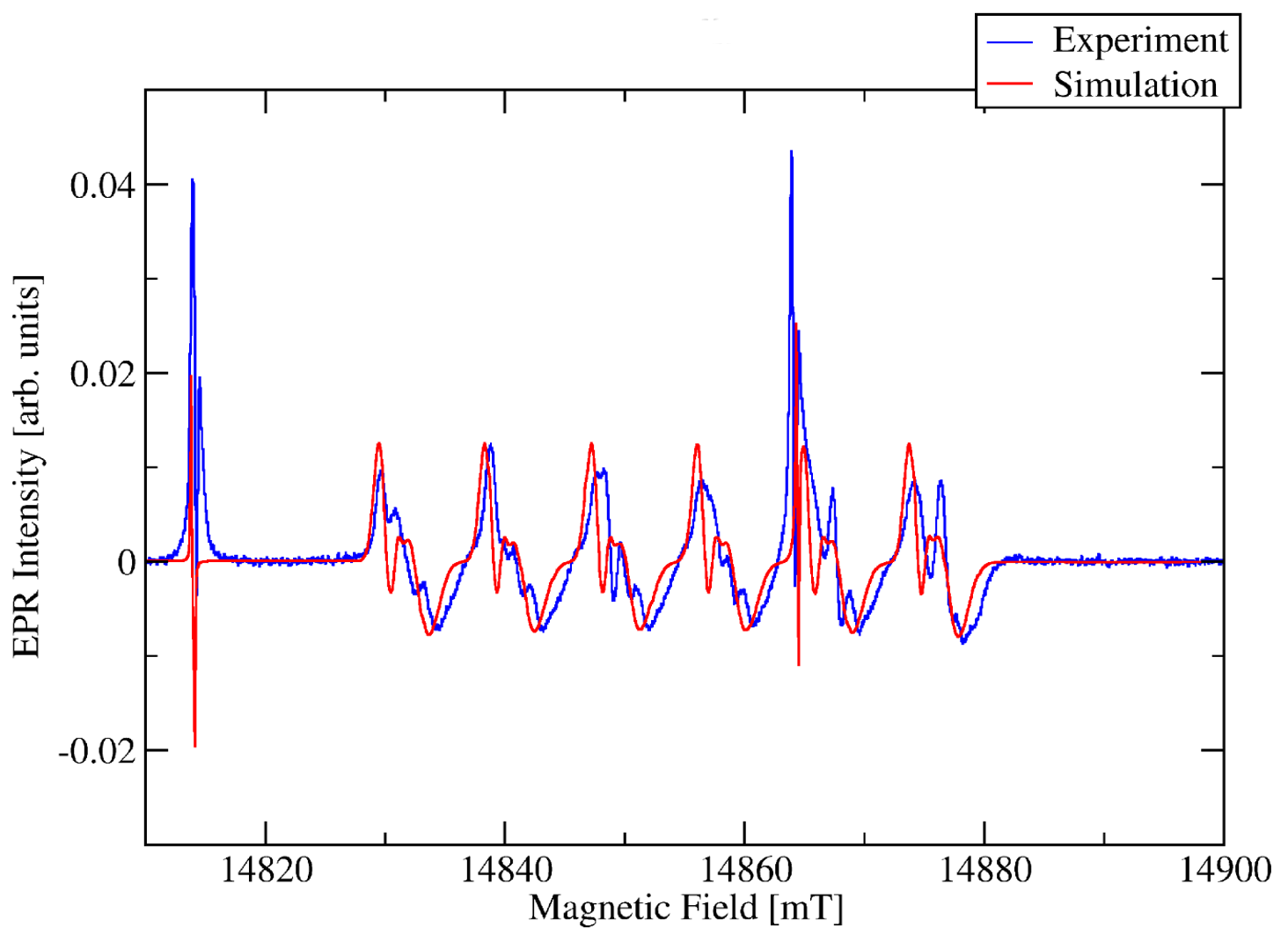

Supplement: Figure S7 — 416.0 GHz EPR spectrum of wild-type CsOxOx in 25 mM Imidazole-Cl, pH 7.0. The experimental and simulated spectra are displayed in blue and red, respectively. (TIF) [file pone.0057933.s007.tif]
