# Supplementary material for: Kinetic and Spectroscopic Studies of Bicupin Oxalate Oxidase and Putative Active Site Mutants
Source: PLoS One. 2013 Mar 1;8(3):e57933. doi: 10.1371/journal.pone.0057933 (PMC3585803; doi:10.1371/journal.pone.0057933)
Supplement: Table S1 — Primers used in the construction of wild type CsOxOx and CsOxOx mutants. (DOCX) [file pone.0057933.s008.docx]

| Enzyme | Type | Primer sequence |
| --- | --- | --- |
| CsOxOx^a^ | Forward | 5’-TAT CT**C** **TCG** **AG**A AAA GAC GCC CCA CCG GCA ACG-3’ |
| CsOxOx^b^ | Reverse | 5’-CCC GT**T** **CTA** **GA**T TAA TCT GAG GCG ACA ACG AAT GC-3’ |
| D241A | Forward | 5’-GAC GGA ACG TTC GCG GCC TCA AAT CAG TTC-3’ |
| D241A | Reverse | 5’-GGC CGC GAA CGT TCC GTC AGG GAA AAT CAG C-3’ |
| D241S | Forward | 5’-GAC GGA ACG TTC TCT GCC TCA AAT CAG TTC-3’ |
| D241S | Reverse | 5’-GGC AGA GAA CGT TCC GTC AGG GAA AAT CAG C-3’ |
| A242E | Forward | 5’-GAC GGA ACG TTC GAT GAG TCA AAT CAG TTC ATG -3’ |
| A242E | Reverse | 5’-TGA CTC ATC GAA CGT TCC GTC AGG GAA AAT CAG C-3’ |
| DASN241-4SENS | Forward | 5’-AGC GAG AAC AGC CAG TTT ATG ATT ACT GAT TGG CTG GC-3’ |
| DASN241-4SENS | Reverse | 5’-AAA CTG GCT GTT CTC GCT GAA GGT GCC GTC AGG G-3’ |
| R169K | Forward | 5’-ACA ATC AAG GAG CTC CAT TGG CAC AAC ACC-3’ |
| R169K | Reverse | 5’-CCA ATG GAG CTC CTT GAT TGT ACC AGC CTC-3’ |
| R349K | Forward | 5’-CCG GGC GCC ATG AAG GAG CTA CAT TGG CAC-3’ |
| R349K | Reverse | 5’-TAG CTC CTT CAT GGC GCC CGG CTC AAC GGT-3’ |

^a^XhoI restriction site engineered (shown in bold)

^b^XbaI restriction site engineered (shown in bold)
